# Polymorphisms in *XPC*, *XPD*, *XRCC1*, and *XRCC3 *DNA repair genes and lung cancer risk in a population of Northern Spain

**DOI:** 10.1186/1471-2407-7-162

**Published:** 2007-08-16

**Authors:** M Felicitas López-Cima, Patricia González-Arriaga, Laura García-Castro, Teresa Pascual, Manuel G Marrón, Xose S Puente, Adonina Tardón

**Affiliations:** 1 Departamento de Medicina, Facultad de Medicina, Unidad de Epidemiología Molecular del Instituto Universitario de Oncología, Universidad de Oviedo, 33006 Oviedo, Spain; 2 Sección de Neumología, Hospital de Cabueñes, Gijón, Spain; 3 Departamento de Bioquímica y Biología Molecular, Universidad de Oviedo, 33006 Oviedo, Spain

## Abstract

**Background:**

Polymorphisms in DNA repair genes have been associated to repair DNA lesions, and might contribute to the individual susceptibility to develop different types of cancer. Nucleotide excision repair (NER), base excision repair (BER), and double-strand break repair (DSBR) are the main DNA repair pathways. We investigated the relationship between polymorphisms in two NER genes, *XPC *(poly (AT) insertion/deletion: PAT-/+) and *XPD *(Asp312Asn and Lys751Gln), the BER gene *XRCC1 *(Arg399Gln), and the DSBR gene *XRCC3 *(Thr241Met) and the risk of developing lung cancer.

**Methods:**

A hospital-based case-control study was designed with 516 lung cancer patients and 533 control subjects, matched on ethnicity, age, and gender. Genotypes were determined by PCR-RFLP and the results were analysed using multivariate unconditional logistic regression, adjusting for age, gender and pack-years.

**Results:**

Borderline association was found for *XPC *and *XPD *NER genes polymorphisms, while no association was observed for polymorphisms in BER and DSBR genes. *XPC PAT+/+ *genotype was associated with no statistically significant increased risk among ever smokers (OR = 1.40; 95%CI = 0.94–2.08), squamous cell carcinoma (OR = 1.44; 95%CI = 0.85–2.44), and adenocarcinoma (OR = 1.72; 95%CI = 0.97–3.04). *XPD *variant genotypes (*312Asn/Asn *and *751Gln/Gln*) presented a not statistically significant risk of developing lung cancer (OR = 1.52; 95%CI = 0.91–2.51; OR = 1.38; 95%CI = 0.85–2.25, respectively), especially among ever smokers (OR = 1.58; 95%CI = 0.96–2.60), heavy smokers (OR = 2.07; 95%CI = 0.74–5.75), and adenocarcinoma (OR = 1.88; 95%CI = 0.97–3.63). On the other hand, individuals homozygous for the XRCC1 *399Gln *allele presented no risk of developing lung cancer (OR = 0.87; 95%CI = 0.57–1.31) except for individuals carriers of *399Gln/Gln *genotype and without family history of cancer (OR = 0.57; 95%CI = 0.33–0.98) and no association was found between *XRCC3 *Thr241Met polymorphism and lung cancer risk (OR = 0.92; 95%CI = 0.56–1.50), except for the *241Met/Met *genotype and squamous cell carcinoma risk (OR = 0.47; 95%CI = 0.23–1.00).

**Conclusion:**

In conclusion, we analysed the association between *XPC*, *XPD*, *XRCC1*, and *XRCC3 *polymorphisms and the individual susceptibility to develop lung cancer in the Spanish population, specifically with a highly tobacco exposed population. We attempt to contribute to the discovery of which biomarkers of DNA repair capacity are useful for screening this high-risk population for primary preventing and early detection of lung cancer.

## Background

Lung cancer is the most common cancer in the world, in 2002 there were 1.35 million new cases, representing 12.4% of total cancers. It was also the most common cause of death from cancer, with 1.18 million global deaths, representing 17.6% of the total deaths from cancer. Almost half (49.9%) of the cases occur in the developing countries of the world [1]. In Spain, lung cancer is the main cancer in men, accounting for 16,628 deaths in 2004 [2].

Although cigarette smoking is the major cause of lung cancer, only a small fraction of smokers develop this disease, suggesting that other causes, including genetic susceptibility, might contribute to the variation in individual lung cancer risk [3,4]. This genetic susceptibility may result from inherited polymorphisms in the genes involved in carcinogen metabolism and DNA damage repair [5-7]. DNA repair systems play a critical role protecting the genome from insults caused by carcinogenic agents, such as those found in tobacco smoke [8]. Until now, more than a hundred proteins implicated in DNA repair have been found in human cells. These proteins are implicated in four major DNA repair pathways, including nucleotide excision repair (NER), base excision repair (BER), double-strand break repair (DSBR) and mismatch repair (MMR) [9,10].

Polymorphisms affecting the coding sequence of a gene are very common in the population, and many of them result in changes that alter protein function [11]. In this sense, the completion of the human genome sequence has allowed the identification of numerous polymorphisms in DNA repair genes, and many of them have been shown to contribute to genetic instability and error accumulation due to reduced protein activity. The gene encoding the NER protein XPC constitutes an excellent example, because a relationship between polymorphism and altered gene function has been established.

In a previous report, we have shown that individuals homozygous for the *XPC *PAT polymorphism have an increased risk of developing lung cancer [12]. Nevertheless, PAT polymorphism in the *XPC *gene has been associated with an increased risk of developing different types of cancer, including smoking-related cancers [13-15] or melanoma [16]. Polymorphisms in other DNA repair NER genes have also been associated with individual susceptibility to develop cancer, including the gene encoding XPD. The presence of the variant alleles *312Asn *and *751Gln *of *XPD *have been associated with relatively high risk of lung cancer in Caucasian [17-20] and Asian [21-24] populations and a recent meta-analysis concludes that the variant genotypes *312Asn/Asn *and *751Gln/Gln *are associated with a statistically significant lung cancer risk in the Caucasian population [25]. Moreover, several studies have carried out combined analysis between lung cancer risk and polymorphisms in different NER genes including *XPC *and *XPD *[19,26]. Functional studies in humans have shown that common polymorphisms in NER genes can modify the capacity to repair DNA [27-29], and epidemiologic studies have supported their role in the pathogenesis of smoking-related cancers [7,30].

BER genes play a key role by removing DNA damage from oxidation, deamination, and ring fragmentation [31] and exposure to tobacco smoking induces oxidative damage by generation of reactive oxygen species (ROS) [32]. Therefore, polymorphisms in BER genes may be associated with lung cancer. The association between the XRCC1 Arg399Gln polymorphism, resulting from a guanine to adenine nucleotide change, and lung cancer risk has been evaluated in a number of epidemiological studies [19,20,33-39]. A recent meta-analysis including 7385 cases and 9381 controls showed that 399Gln/Gln genotype was associated with an increased risk of lung cancer among Asians but not among Caucasians [37]. A multicenter study conducted in Europe concluded that this polymorphism was not associated with lung cancer risk [34].

Finally, DSBR pathway is the responsible for repairing double-strand breaks. These result from exogenous agents such as ionizing radiation or environmental carcinogens, including those present in tobacco smoke and from endogenously generated ROS. They can also be produced when DNA replication encounter DNA single-strand breaks or other types of lesion [40]. XRCC3, which participates in DNA double-strand break via homologous recombinational repair, presents a non-conservative Thr241Met substitution in exon 7. Until now, there are several conflicting reports on the association between this polymorphism and lung cancer risk in the Caucasian population [19,20,38,41-43].

In order to examine if genetic polymorphisms in DNA repair genes implicated in NER, BER and DSBR pathways are associated with lung cancer risk, we have studied five polymorphisms in four genes (*XPC, XPD*, *XRCC1*, *XRCC3*) in 516 cases and 533 controls of a Caucasian population of Northern Spain, historically highly exposed to tobacco.

## Methods

### Study population

The CAPUA study (Cáncer de Pulmón en Asturias) is a hospital-based case-control study conducted in the "Unidad de Epidemiología Molecular del Cáncer, Instituto Universitario de Oncología" of Universidad de Oviedo. Patients were recruited in two main hospitals following an identical protocol from October 2000 to April 2005. Eligible cases were incident cases of histologically confirmed lung cancer between 30 and 85 years of age and residents in the geographical area of each participating hospital for at least six months before diagnosis. Patients with primary cancer other than lung cancer occurring in the last 5 years were excluded. Controls were selected from patients admitted to participating hospitals for diagnoses believed to be unrelated to the exposures of interest, individually matched to the cases on ethnicity, gender and age (± 5 years). The main specific pathologies of the final controls selected were: 41.1% inguinal and abdominal hernias (ICD-9: 550–553), 32.5% injuries (ICD-9: 800–848, 860–869, 880–897), 8.8% appendicitis (ICD-9: 540), and 13.3% intestinal obstructions (ICD-9: 560, 569, 574). The study was approved by the ethical committee of the hospitals, and written consent was obtained from each participant.

### Data collection

Information on known or potential risk factors for lung cancer was collected personally through computer-assisted questionnaires by trained interviewers during the first hospital admission for diagnosis. Structured questionnaires collected information on sociodemographic characteristics, recent and prior tobacco use, environmental exposure (air pollution, environmental tobacco smoking (ETS)), diet, personal and family history of cancer, and occupational history from each participant. A total of 93.8% eligible cases and 98.5% of eligible controls agreed to participate in the study and were interviewed. Of the 759 cases and 593 controls interviewed, 741 (97.6%) cases and 556 (93.8%) controls provided a blood or buccal cell sample for DNA extraction. Seventeen individuals (five cases and twelve controls) were excluded because of low amounts of DNA. 37 individuals (twenty six cases and eleven controls) with missing information in the questionnaires and 194 cases without matched controls were also excluded from the analyses. Thus, the final study population available for analysis was 516 cases and 533 controls, all of whom were Caucasian.

### Tobacco exposure information

Participants were defined as never smokers if they had not smoked >100 cigarettes in their lifetime and ever smokers otherwise. Ever smokers were further classified as current smokers if they had smoked at least one cigarette per day for 6 months or longer. Individuals who had smoked regularly but who had stopped smoking at least 1 year before the interview were defined as former smokers. ETS exposure was quantified determining the source, intensity, and duration of exposure during childhood and adulthood [44]. Smoking intensity (pack-years, PY) was defined as the number of packs of cigarettes smoked per day multiplied by the number of years smoking. We categorized the subjects as light (≤ 16.45 PY), moderate (> 16.45–53 PY), or heavy (> 53 PY) smokers based on the quartiles of cumulative tobacco consumption among the control group.

### Genotype analysis

Laboratory personnel were blinded to case and control status. Genomic DNA was extracted from peripheral blood samples (96.5% of total) or exfoliated buccal cells (3.5% of total) as previously described [45]. For quality control, genotyping was repeated randomly in at least 5% of the samples, and two of the authors independently reviewed all results. A quality control of 50 blood and mouthwash samples from the same participants ensured the reliability of genotyping results of mouthwash samples. In both quality controls no differences were found. Polymorphisms studied are shown in Table 1. To determine the *XPC *PAT polymorphism, intron 9 of the *XPC *gene was amplified by polymerase chain reaction (PCR) using the oligonucleotides shown in Table 2 (primers and conditions were previously described [12]). The polymorphisms in *XPD *exon 10 (rs1799793), *XPD *exon 23 (rs13181), *XRCC1 *(rs25487) and *XRCC3 *(rs861539) were analysed by PCR combined with restriction fragment length polymorphism (RFLP). Details of PCR primers and cycle conditions used are shown in Table 2. In the case of the *XRCC3 *gene, the reverse primer was specially designed to introduce the recognition site of the restriction enzyme *Nco*I by replacing a G with a C (lower case). PCR was performed in a 10 μl mixture containing 20 ng of genomic DNA, 0.25 mM each dNTP, 0.5 units of *Taq *polymerase (Biotools), and 10 pmol of each primer in 1 × PCR buffer. For the amplification of *XPD *exon 10, dimethylsulfoxide was added to the reaction at a final concentration of 3%. PCR products were digested overnight with the indicated restriction enzyme at 37°C. DNA fragments were resolved on agarose gels and stained with ethidium bromide (restriction enzyme and fragments sizes are shown in Table 1). To verify that the data obtained by RFLP was coincident with the allele sequence, representative fragments were further purified for PCR-directed sequencing to confirm the different polymorphisms (data not shown).

**Table 1:** Details of RFLPs studied and fragments sizes

**Repair**	**Gene**	**exon/intron**	**Enzyme**	Polymorphism*		**Genotype**	**Fragment sizes (bp)**
NER	*XPC*	intron 9	-	insertion 83 pb	-	*PAT *-/- *PAT *+/+	266 344
	*XPD*	exon 10	*Sty*I	G23591A (rs 1799793)	Asp312Asn	*Asp/Asp* *Asn/Asn*	244+507 33+244+474
	*XPD*	exon 23	*Pst*I	A35931C (rs 13181)	Lys751Gln	*Lys/Lys* *Gln/Gln*	146+290 63+146+227
BER	*XRCC*1	exon 10	*Msp*I	G28152A (rs 25487)	Arg399Gln	*Arg/Arg* *Gln/Gln*	132+278+461 278+593
DSBR	*XRCC*3	exon 7	*Nco*I	C18067T (rs 861539)	Thr241Met	*Thr/Thr* *Met/Met*	136 39+97

* Reference SNP accession numbers are indicated for each polymorphism

**Table 2:** Details of PCR conditions for polymorphism analysis

**Product**	**Primer sequence**	**Size (bp)**	**PCR Conditions**
XPC (PAT)	(F) TAG CAC CCA GCA GTC AAA G (R) TGT GAA TGT GCT TAA TGC TG	266/344	30 cycles: 94°C 30s, 58°C 30s, 72°C 30s
XPD exon 10	(F) CTG TTG GTG GGT GCC CGT ATC TGT TGG TCT (R) TAA TAT CGG GGC TCA CCC TGC AGC ACT TCC T	751	40 cycles: 94°C 30s, 65°C 30s, 72°C 1 min
XPD exon 23	(F) GCC CGC TCT GGA TTA TAC G (R) CTA TCA TCT CCT GGC CCC C	436	30 cycles: 94°C 30s, 64°C 30s, 72°C 45s
XRCC1	(F) CAG TGG TGC TAA CCT AAT C (R) AGT AGT CTG CTG GCT CTG G	871	30 cycles: 94°C 30s, 64°C 30s, 72°C 1 min
XRCC3	(F) GCC TGG TGG TCA TCG ACT C (R) CAG GGC TCT GGA AGG CAC TGC TCA GCT CAC GCA cC	136	30 cycles: 94°C 30s, 65°C 30s, 72°C 1 min

### Statistical analysis

Tests for Hardy-Weinberg equilibrium among controls were conducted using observed genotype frequencies and a χ^2 ^test with one degree of freedom. Univariate analysis was first performed to compare the distribution of age and gender and the frequencies of alleles and genotypes. The differences in the distribution between cases and controls were tested using the χ^2^, Fisher exact, and Mann-Whitney U-test, where appropriate. The crude odd ratios (ORs) were calculated by Wolf's method [46]. Multivariate unconditional logistic regression analysis with adjustment for age, gender, and pack-years was performed to calculate adjusted ORs and 95% confidence intervals (CIs). Gene-gene and gene-environment interactions were estimated by the logistic regression model, which included an interaction term as well as variables for exposure (smoking), genotypes (*XPC*, *XPD*, *XRCC1 *or *XRCC3*) and potential confounders (age and gender). All statistical analyses were performed with STATA version 8 software.

The sample size of our study for an allele frequency between 29–32% is enough to detect ORs greater than 1.38 with more than 90% power assuming a log-additive model. For allele frequencies of 40%, the power to detect an OR of 1.28 is 79%. For allele frequencies between 30–40% as observed for polymorphisms analysed in this study, the power to detect an OR greater than 2.00 for the interaction gen-gen is more than 90%. Allele frequencies of controls were calculated using following formula (example genotypes AA, AB, BB): Allele B frequency = [number genotypes AB + 2 × (number genotypes BB)]/[2 × (number genotypes AA + number genotypes AB + number genotypes BB)].

## Results

### Subject characteristics

The analysis included 516 lung cancer cases and 533 controls from the Caucasian population of Asturias, Northern Spain. The distributions of age, gender, smoking history, family history of cancer, and histological type for the cases among the study subjects are summarized in Table 3. There were no statistically significant differences among cases and controls in terms of mean age and gender distributions, suggesting that the frequency matching was adequate. There is only a never smoker case of lung cancer without ETS exposure and there were more current smokers (53.2% *vs*. 39.9%) and more heavy smokers (62.85 *vs*. 40.41 number of pack-years, PY) in the study cases than in the control group (*P *< 0.001). There is a statistically significant difference between cases and controls regarding type of tobacco smoked, 75.3% of cases were smokers of black tobacco (black smokers), exclusively. Histologically, squamous cell carcinoma (40.3%) and adenocarcinoma (29.5%) are the main types of lung cancer presented.

**Table 3:** Characteristics of lung cancer cases and control patients in a Spanish population

Variable	Cases (n = 516) n (%)	Controls (n = 533) n (%)	*P*^a^
Gender			
Male	456 (88.4)	460 (86.3)	
Female	60 (11.6)	73 (13.7)	0.314
			
Age (yrs), mean (SD)	64.79 (10.99)	63.54 (11.33)	0.090
			
Smoking Status			
Never	1 (0.2)	4 (0.7)	
ETS exposed	35 (6.8)	141 (26.45)	
Ever	480 (93.0)	388 (73.0)	< 0.001
Former	222 (46.8)	224 (60.1)	
Current	252 (53.2)	149 (39.9)	< 0.001
			
Type of tobacco			
Only black	359 (75.3)	223 (60.6)	
Only blond	47 (9.8)	84 (22.8)	
Both	71 (14.9)	61 (16.6)	< 0.001
			
Pack-years^b^, mean (SD)	62.85 (36.18)	40.41 (38.95)	< 0.001
			
Family history of cancer			
No	272 (57.3)	317 (62.2)	
Lung cancer	57 (12.0)	35 (6.9)	
Other cancer	146 (30.7)	158 (31.0)	0.019
			
Histological type			
Squamous cell carcinoma	208 (40.3)		
Adenocarcinoma	152 (29.5)		
Small cell carcinoma	83 (16.1)		
Large cell carcinoma	16 (3.1)		
Non-differentiated	39 (7.6)		
Others	7 (1.3)		
Clinical diagnosis	2 (0.4)		
Missing	9 (1.7)		

^a ^Two-sided χ^2 ^test and Mann-Whitney where appropriate

^b ^Pack-years for ever smokers

We have determined the frequency of 5 polymorphisms in 4 different genes implicated in DNA damage repair (*XPC *PAT, *XPD *Asp312Asn, *XPD *Lys751Gln, *XRCC1 *Arg399Gln, and *XRCC3 *Thr241Met) in lung cancer patients and matched controls in order to evaluate their association with the risk of lung cancer. The genotype distribution for all the SNPs studied was consistent with Hardy-Weinberg equilibrium. In the multivariate logistic regression model, there was no evidence for any interaction between variant genotypes and smoking (data not shown).

### Analysis of the *XPC PAT *genotype

The frequency of the *XPC PAT+ *allele was 0.431 in study cases and 0.401 in controls. The frequency of the *PAT+/+ *genotype was higher in the study cases (19.6%) than in controls (15.8%), although not significantly (*P *= 0.260) (Table 4). When we analysed the association between *XPC *genotypes and lung cancer risk, we found that those individuals homozygous for the *PAT+ *allele presented a not statistically significant higher risk of lung cancer (adjusted OR = 1.28; 95% CI = 0.85–1.92, *P *= 0.229).

**Table 4:** Analysis of polymorphisms and lung cancer risk estimates

**Repair**	**Gen**	**SNP**	**Genotype**	**Cases n (%)**	**Controls n (%)**	**Adjusted^a ^OR [95% IC]**	*P*	***P *trend**
**NER**	***XPC***	PAT	*-/-*	172 (33.3)	190 (35.6)	Reference		
			*+/-*	243 (47.1)	259 (48.6)	1.08 [0.79–1.47]	0.627	
			***+/+***	**101 (19.6)**	**84 (15.8)**	**1.28 [0.85–1.92]**	0.229	0.246
	
	***XPD***	Asp312Asn	*Asp/Asp*	240 (46.5)	260 (48.8)	Reference		
			*Asp/Asn*	221 (42.8)	230 (43.1)	1.01 [0.76–1.35]	0.934	
			***Asn/Asn***	**55 (10.7)**	**43 (8.1)**	**1.52 [0.91–2.51]**	0.106	0.232
		Lys751Gln	*Lys/Lys*	222 (43.0)	243 (45.6)	Reference		
			*Lys/Gln*	237 (45.9)	240 (45.0)	1.12 [0.84–1.50]	0.437	
			***Gln/Gln***	**57 (11.1)**	**50 (9.4)**	**1.38 [0.85–2.25]**	0.193	0.181

**BER**	***XRCC*1**	Arg399Gln	*Arg/Arg*	222 (43.0)	217 (40.7)	Reference		
			*Arg/Gln*	219 (42.5)	234 (43.9)	0.86 [0.63–1.16]	0.320	
			Gln/Gln	**75 (14.5)**	**82 (15.4)**	**0.87 [0.57–1.31]**	0.500	0.672

**DSBR**	***XRCC*3**	Thr241Met	*Thr/Thr*	168 (41.7)	178 (41.0)	Reference		
			*Thr/Met*	185 (45.9)	196 (45.2)	1.06 [0.76–1.49]	0.724	
			***Met/Met***	**50 (12.4)**	**60 (13.8)**	**0.92 [0.56–1.50]**	0.898	0.898

^a ^Adjusted by age, gender and cumulative tobacco consumption (in pack-years: ≤ 16.45, > 16.45–53 and > 53)

Stratified analysis for smoking status showed that the *XPC PAT+/+ *genotype was associated with a not statistically significant increased risk among ever smokers (adjusted OR = 1.40; 95% CI = 0.94–2.08, *P *= 0.100) and heavy black smokers (adjusted OR = 1.55; 95% CI = 0.62–3.87, *P *= 0.350) and stratification for histological type revealed that the variant *PAT+/+ *genotype was associated with a not statistically significant increased risk of developing squamous cell carcinoma (adjusted OR = 1.44; 95% CI = 0.85–2.44, *P *= 0.175) and adenocarcinoma (adjusted OR = 1.72; 95% CI = 0.97–3.04, *P *= 0.064) [see Additional file 1].

### Analysis of the Asp312Asn and Lys751Gln polymorphisms in the *XPD *gene

Analysis of the two most common polymorphisms in the *XPD *gene, Asp312Asn in exon 10 and Lys751Gln in exon 23, revealed that the two polymorphisms were in linkage disequilibrium with 20% of discrepancies, which is in agreement with previous reports [17,18,47,48]. Due to this linkage between both polymorphisms, the OR observed for each allele, either global or stratified, were very similar. The frequencies of the *312Asn *and *751Gln *alleles were 0.321 and 0.340 among study cases and 0.296 and 0.319 among controls, respectively. Genotype distribution and calculated ORs were very similar for both polymorphisms (Table 4), although a higher risk was observed for the Asp312Asn polymorphism. Those individuals homozygous for the *XPD *polymorphisms (*312Asn/Asn *and *751Gln/Gln*) presented a not statistically significant higher risk of developing lung cancer (adjusted OR = 1.52; 95% CI = 0.91–2.51, *P *= 0.106; adjusted OR = 1.38; 95% CI = 0.85–2.25, *P *= 0.193, respectively).

Stratified analysis showed that the *312Asn/Asn *genotype was associated with a not statistically significant increased risk among ever smokers (adjusted OR = 1.58; 95% CI = 0.96–2.60, *P *= 0.074) and heavy smokers (adjusted OR = 2.07; 95% CI = 0.74–5.75, *P *= 0.165), as well as with an increased risk of developing adenocarcinoma (adjusted OR = 1.88; 95% CI = 0.97–3.63, *P *= 0.061) [see Additional file 2].

### Analysis of the Arg399Gln polymorphism in the *XRCC1 *gene

The frequency of the *XRCC1 399Gln *allele was 0.358 in study cases and 0.373 in controls. The frequency of the *Gln/Gln *genotype was lower in the study cases (14.5%) than in controls (15.4%), although this was not statistically significant (*P *= 0.744). Individuals homozygous for the *399Gln *allele presented no risk of developing lung cancer (adjusted OR = 0.87; 95% CI = 0.57–1.31, *P *= 0.500) (Table 4). Stratified analysis for selected variables confirmed the absence of association except for individuals carriers of *399Gln/Gln *genotype and without family history of cancer (adjusted OR = 0.57; 95% CI = 0.33–0.98, *P *= 0.042), which showed a statistically significant protective effect. This genotype was also associated with a not statistically significant increased risk among light smokers (adjusted OR = 1.62; 95% CI = 0.47–5.56, *P *= 0.444), but decreased risk for moderate smokers (adjusted OR = 0.67; 95% CI = 0.36–1.24, *P *= 0.203) [see Additional file 3].

### Analysis of the Thr241Met polymorphism in the *XRCC3 *gene

The frequency of the *XRCC3 241Met *allele was 0.354 in study cases and 0.364 in controls. The frequency of the *241Met/Met *genotype in *XRCC3 *was similar in the study cases (12.4%) and in controls (13.8%), and no association was found between *XRCC3 *Thr241Met polymorphism and lung cancer risk (adjusted OR = 0.92; 95% CI = 0.56–1.50, *P *= 0.898) (Table 4). Stratified analysis for selected variables confirmed the absence of association except for the *241Met/Met *genotype and squamous cell carcinoma risk (adjusted OR = 0.47; 95% CI = 0.23–1.00, *P *= 0.049) showing a protective effect [see Additional file 4].

### Combined analysis of polymorphisms in DNA repair genes and lung cancer

Finally, in order to test whether individual polymorphisms in DNA repair genes might interact and modify the risk of developing lung cancer, ORs were estimated for each pair of the studied polymorphisms (*XPC *PAT, *XPD *Asp312Asn, *XPD *Lys751Gln, *XRCC1 *Arg399Gln and *XRCC3 *Thr241Met). Our results show an interaction between *XPC*/*XPD, XPC*/*XRCC3 *and *XPD*/*XRCC3 *polymorphisms (Table 5). In fact, individuals with genotypes *XPC PAT(+/+)/XPD 751Lys/Gln *or *XPC PAT(+/+)/XPD 751Gln/Gln *showed a 1.63-fold (CI = 0.89–2.98), *P *= 0.111, and 2.25-fold (CI = 0.83–6.13), *P *= 0.202, higher risk of lung cancer, respectively, when compared with homozygous carriers of the wild type allele of both polymorphisms (*XPC PAT(-/-)/XPD 751Lys/Lys*). Furthermore, despite the fact that the polymorphism in *XRCC3 *didn't alter the overall risk of developing lung cancer when studied independently, when this polymorphism was combined with those studied in *XPC *or *XPD*, we observed an interaction between these polymorphisms. Individuals with the *XPC PAT(+/+)/XRCC3 241Met/Met *or *XPD 751Gln/Gln/XRCC3 241Met/Met *genotypes showed a not significant higher risk of developing lung cancer 3.06 (CI = 0.91–10.30) (*P *= 0.071) and 2.66 (CI = 0.74–9.62) (*P *= 0.135) respectively.

**Table 5:** Combined analysis for *XPC*, *XPD *and *XRCC3 *polymorphisms and lung cancer risk estimates

**Genotype 1**	**Genotype 2**	**Cases n(%)**	**Controls n(%)**	**Adjusted^a ^OR [95% CI]**	***P***	***P*_interaction_**
XPC PAT	XPD Lys751Gln					
-/-	*Lys/Lys*	72 (13.9)	84 (15.8)	1.00 (reference)		
**+/+**	*Lys/Lys*	36 (7.0)	38 (7.1)	1.10 [0.59–2.06]	0.971	
**+/+**	*Lys/Gln*	49 (9.5)	37 (6.9)	1.63 [0.89–2.98]	0.111	
**+/+**	***Gln/Gln***	**16 (3.1)**	**9 (1.7)**	**2.25 [0.83–6.13]**	0.202	0.202

XPC PAT	XRCC3 Thr241Met					
-/-	*Thr/Thr*	49 (12.2)	60 (13.8)	1.00 (reference)		
**+/+**	*Thr/Thr*	36 (8.9)	31 (7.1)	1.22 [0.61–2.43]	0.578	
**+/+**	*Thr/Met*	34 (8.4)	27 (6.2)	1.41 [0.68–2.92]	0.351	
**+/+**	***Met/Met ***	**12 (3.0)**	**6 (1.4)**	**3.06 [0.91–10.30]**	0.071	0.174

XPD Lys751Gln	XRCC3 Thr241Met					
*Lys/Lys*	*Thr/Thr*	83 (20.6)	89 (20.5)	1.00 (reference)		
***Gln/Gln***	*Thr/Thr*	17 (4.2)	17 (3.9)	0.98 [0.41–2.35]	0.968	
***Gln/Gln***	*Thr/Met*	20 (5.0)	18 (4.1)	1.59 [0.70–3.59]	0.264	
***Gln/Gln***	***Met/Met ***	**10 (2.5)**	**5 (1.2)**	**2.66 [0.74–9.62]**	0.135	0.261

^a ^Adjusted by age, gender and cumulative tobacco consumption (in pack-years: ≤ 16.45, > 16.45–53 and > 53)

## Discussion

In this study, we have examined whether polymorphisms in four DNA repair genes involved in the nucleotide excision (NER), base excision (BER), and double-strand break (DSBR) DNA repair pathways are implicated in the development of lung cancer in a Caucasian population from Asturias, Northern Spain. Our results support that polymorphisms in two different NER genes (*XPC *and *XPD*) increased the risk of developing lung cancer, so individuals homozygous for the *XPC PAT+*, *XPD 312Asn *or *XPD 751Gln *alleles have a higher risk of developing lung cancer (ORs 1.28, 1.52 and 1.38, respectively). This association was particularly important for ever smokers and patients with adenocarcinomas. On the other hand, no association was found between two genes that participate in the BER and DSBR repair processes (*XRCC1 *and *XRCC3*) and the risk of lung cancer. Additionally, interaction between *XPC *and *XPD *polymorphisms showed an increased risk of lung cancer (OR = 2.25). Similarly, interactions between *XPC*/*XRCC3 *and *XPD*/*XRCC3 *were observed, suggesting that coordination between both repair systems might contribute to the individual susceptibility to develop cancer.

Our study has several strengths, including high participation of eligible cases (rate 93.8%), quite large sample size from a homogeneous population of same ancestors (516 cases and 533 controls) and the fact that all our control subjects were under Hardy-Weinberg equilibrium. Nevertheless all our cases were pathology confirmed and finally we applied a severe quality control from genotyping. The main limitations of our study were hospital-based subjects, recall bias due to the fact that information on smoking exposure was obtained retrospectively, and especially possible false positive associations, due to multiple comparisons made, we cannot exclude the possibility that some of these associations may represent chance finding, because the power to detect interactions was limited. On the other hand, we have to bear in mind that 26% of controls were ETS exposed which could lead to underestimate our results. To limit selection bias, we carefully selected controls from patients admitted for various diagnoses that were thought to be unrelated to exposures of interest. Nevertheless, a recent paper from Campbell *et al*. [49] reported that European populations may display various levels of genetic substructure which may lead to false positive associations due to population stratification. In our study, we controlled for this possibility by matching individuals on the basis of European ancestry.

We have previously shown that the PAT+ allele is in complete linkage disequilibrium with the intron 11 A-allele [12], reflecting the *XPC *haplotype (*PAT+/939Gln/intron 11 A*) with a reduced ability to repair DNA lesions and an increased risk of developing lung cancer. Previous functional analysis has shown that cells with the *A/A *genotype at the splice acceptor site in intron 11 have a higher frequency of deletion of exon 12 [50], suggesting that this mechanism might contribute to the reduced ability of individuals with this genotype to repair DNA lesions. Nevertheless, the effect of the Lys939Gln polymorphism on the biochemical activity of XPC is still under investigation.

Several reports have shown that polymorphisms in the *XPC *gene increase the risk of different tumor types, including smoking-related cancers and cutaneous melanoma [13-16,51,52]. For lung cancer, the number of studies is still very limited. A recent study carried out in an Asiatic population of 432 cases and 432 controls was unable to find any association between the *XPC *PAT polymorphism and the risk of developing lung cancer [53]. However, other reports studying the exon 15 polymorphism in Danish and Chinese populations have found an increased risk for developing lung cancer for the *939Gln *allele [26,54], similar to our results.

The *312Asn *and *751Gln *alleles in the *XPD *gene have been associated with a reduced capacity to repair BPDE and UV-induced damage in host cell reactivation assays [48,55,56] and with a higher DNA adduct, chromosomal aberrations, and single-strand breaks level which is interpreted as lower repair efficiency [27,28,57-59]. Our results confirm an association between these polymorphisms and the risk of developing lung cancer, and extend previous findings [17-22,24,25,60].

Our results for the stratified analysis are supported by biological evidence. Tobacco smoke increases the risk of lung cancer and increases the risk for all histological types of this cancer, including adenocarcinoma [61]. Our results showed higher risk for adenocarcinoma, although the reason for the observed histology-dependent difference in the genetic effect conferred by these polymorphisms is unknown, being perhaps a bit too hypothetical, it may be attributable to differences in the carcinogenesis pathways among the histological types of lung cancer. Various lines of evidence have suggested that the histological type of lung cancer may be determined by the particular initiating agent to which an individual is exposed [62,63], which need to be verified in further studies. Therefore, genetic factors involved in susceptibility could be different between the histological subtypes of lung cancer [21,24,53].

Contrary to the results observed with polymorphisms in genes that participate in the NER mechanism, the polymorphisms studied in *XRCC1 *and *XRCC3*, implicated in other DNA repair processes such as BER and DSBR, were not associated to the global individual susceptibility to develop lung cancer. Previous studies of XRCC1 Arg399Gln polymorphism have shown contradictory results, several reports have found association with different types of cancer, including colorectal, breast, lung or melanoma [64-70], while other reports have failed to find association with some of these pathologies, or even found a protective effect [71-73]. Our data showed no association between XRCC1 Arg399Gln and lung cancer risk, but *399Gln/Gln *genotype showed a not significant increased risk for light smokers, suggesting any kind of effect modification as Hung et al. concluded for all smoking related cancers [74]. These results fit in studies showing *399Gln *allele may be associated with higher mutagen sensitivity and higher levels of DNA adducts [75] who reported that never smokers carriers of *399Gln *had higher DNA adduct levels than current smokers.

The *XRCC*3 *241Met *allele has previously been associated with less efficient DNA repair [75], as well as an increased number of centrosomes and binucleated cells [76]. However, it has also been shown that the common and the variant *XRCC3 *alleles are functionally equivalent in the double-strand break repair pathway [77], which may explain the lack of association between XRCC3 Thr241Met polymorphism and lung cancer risk shown in several studies [41,42,47]. In the Caucasian population, there are inconclusive and conflicting results: several studies have found an increased risk for non small cell carcinoma and lung cancer [19,43], while other studies have shown a protective effect, once more for non small cell carcinoma and ever smokers [20,38]. Our study showed a statistically significant protective effect for squamous cell carcinoma, but it is difficult to assess the effect of this single common sequence variant because it might not be detectable in population association studies being necessary larger samples.

We have found that polymorphisms in NER genes increase the risk of developing lung cancer, while no association was found between polymorphisms in BER and DSBR genes and lung cancer risk. These results might reflect differences in the etiology of different carcinomas, or a more important role of the NER repair pathways in the development of lung cancer. In this regard, numerous studies have shown that most DNA lesions caused by tobacco-smoke carcinogens are repaired by the NER mechanism [8,78,79], suggesting that this particular cancer could be more susceptible to polymorphisms affecting genes implicated in the NER pathway.

Although the relative risks for individuals carrying the polymorphisms in *XPC *and *XPD *genes are modest (ORs < 1.52), these polymorphisms could account for a large proportion of lung cancers, as they are very common in the population. In fact, between 9% and 16% of individuals are homozygous for the high-risk genotypes (*XPC PAT+/+ *or *XPD 751Gln/Gln*). In this regard, we observed a borderline combined effect between these polymorphisms and the risk of lung cancer, as individuals homozygous for both risk genotypes showed a further increase in the risk of developing lung cancer than that observed for the individual polymorphisms (adjusted OR = 2.25; 95% CI 0.83–6.13, *P *= 0.202). This combined effect of *XPC *and *XPD *polymorphisms could support the hypothesis for this population that changes in genes implicated in the NER repair pathway contribute to the susceptibility of developing lung cancer, and the combination of genotypes with a reduced ability to repair DNA lesions could result in a higher risk of developing this disease.

Similarly, when we combined *XRCC3 241Met/Met *genotype with the *XPC PAT+/+ *or the *XPD 751Gln/Gln *genotypes, an increased risk was observed (Table 5). These results could suggest that the DSBR mechanism might also play a role in the development of lung cancer when combined with certain NER genes genotypes. Indeed, smoking induces a great variety of DNA damage, which must be repaired by more than one repair pathway, being NER the main pathway and DSBR the second, thus the combined occurrence of genetic variants in these two repair pathways might contribute to a greater risk of lung cancer. The approach of using combined analysis of polymorphisms may represent an alternative way of analyzing the overall effect of the different genetic variants as well as the potential joint effect of these genes.

## Conclusion

In conclusion, we analysed the association between *XPC*, *XPD*, *XRCC1*, and *XRCC3 *polymorphisms and the individual susceptibility to develop lung cancer in the Spanish population, specifically with a highly tobacco exposed population. We attempt to contribute to the discovery of which biomarkers of DNA repair capacity are useful for screening high-risk populations for primary preventing and early detection of lung cancer. To further evaluate gene-gene and gene-environment interactions between this polymorphisms and lung cancer risk in our population, a single larger sample with thousands of subjects and tissue-specific biochemical and biological characterizations are required. Finally, higher sample size will be also required to confirm small associations and to evaluate complex interrelationships between genetic variants and smoking type and status.

## Competing interests

The author(s) declare that they have no competing interests.

## Authors' contributions

MFLC carried out the molecular genetic studies and draft the manuscript. PGA participated in the molecular genetic studies. LGC participated in the design of the study, performed the statistical analysis, and revised the manuscript. TP and MGM participated in patient enrollment. XSP participated in the molecular genetic studies. AT conceived of the study, participated in its design and coordination, and revised the manuscript. All authors read and approved the final manuscript.

## Pre-publication history

The pre-publication history for this paper can be accessed here:



## Supplementary Material

Additional file 1 Table 6 – Analysis of *XPC *PAT stratified by selected variables. This table shows the stratified analysis by selected variables of *XPC *PAT polymorphism Click here for file

Additional file 2 Table 7 – Analysis of *XPD *exon 10 stratified by selected variables. This table shows the stratified analysis by selected variables of *XPD *exon 10 (Asp312Asn) polymorphism Click here for file

Additional file 3 Table 8 – Analysis of *XRCC1 *Arg399Gln stratified by selected variables. This table shows the stratified analysis by selected variables of *XRCC1 *Arg399Gln polymorphism Click here for file

Additional file 4 Table 9 – Analysis of *XRCC3 *Thr241Met stratified by selected variables. This table shows the stratified analysis by selected variables of *XRCC3 *Thr241Met polymorphism Click here for file

## References

[B1] Parkin DM, Bray F, Ferlay J, Pisani P (2005). Global cancer statistics, 2002. CA Cancer J Clin.

[B2] CNE (2004). Centro Nacional de Estadística. Defunciones según la causa de muerte en España.

[B3] Tardon A, Lee WJ, Delgado-Rodriguez M, Dosemeci M, Albanes D, Hoover R, Blair A (2005). Leisure-time physical activity and lung cancer: a meta-analysis. Cancer Causes Control.

[B4] Rodriguez V, Tardon A, Kogevinas M, Prieto CS, Cueto A, Garcia M, Menendez IA, Zaplana J (2000). Lung cancer risk in iron and steel foundry workers: a nested case control study in Asturias, Spain. Am J Ind Med.

[B5] Mohrenweiser HW, Jones IM (1998). Variation in DNA repair is a factor in cancer susceptibility: a paradigm for the promises and perils of individual and population risk estimation?. Mutat Res.

[B6] Shields PG, Harris CC (2000). Cancer risk and low-penetrance susceptibility genes in gene-environment interactions. J Clin Oncol.

[B7] Goode EL, Ulrich CM, Potter JD (2002). Polymorphisms in DNA repair genes and associations with cancer risk. Cancer Epidemiol Biomarkers Prev.

[B8] Hoeijmakers JH (1993). Nucleotide excision repair. II: From yeast to mammals. Trends Genet.

[B9] Yu Z, Chen J, Ford BN, Brackley ME, Glickman BW (1999). Human DNA repair systems: an overview. Environ Mol Mutagen.

[B10] Wood RD, Mitchell M, Sgouros J, Lindahl T (2001). Human DNA repair genes. Science.

[B11] (2005). A haplotype map of the human genome. Nature.

[B12] Marin MS, Lopez-Cima MF, Garcia-Castro L, Pascual T, Marron MG, Tardon A (2004). Poly (AT) polymorphism in intron 11 of the XPC DNA repair gene enhances the risk of lung cancer. Cancer Epidemiol Biomarkers Prev.

[B13] Shen H, Sturgis EM, Khan SG, Qiao Y, Shahlavi T, Eicher SA, Xu Y, Wang X, Strom SS, Spitz MR, Kraemer KH, Wei Q (2001). An intronic poly (AT) polymorphism of the DNA repair gene XPC and risk of squamous cell carcinoma of the head and neck: a case-control study. Cancer Res.

[B14] Casson AG, Zheng Z, Evans SC, Veugelers PJ, Porter GA, Guernsey DL (2005). Polymorphisms in DNA repair genes in the molecular pathogenesis of esophageal (Barrett) adenocarcinoma. Carcinogenesis.

[B15] Kietthubthew S, Sriplung H, Au WW, Ishida T (2006). Polymorphism in DNA repair genes and oral squamous cell carcinoma in Thailand. Int J Hyg Environ Health.

[B16] Blankenburg S, Konig IR, Moessner R, Laspe P, Thoms KM, Krueger U, Khan SG, Westphal G, Berking C, Volkenandt M, Reich K, Neumann C, Ziegler A, Kraemer KH, Emmert S (2005). Assessment of 3 xeroderma pigmentosum group C gene polymorphisms and risk of cutaneous melanoma: a case-control study. Carcinogenesis.

[B17] Zhou W, Liu G, Miller DP, Thurston SW, Xu LL, Wain JC, Lynch TJ, Su L, Christiani DC (2002). Gene-environment interaction for the ERCC2 polymorphisms and cumulative cigarette smoking exposure in lung cancer. Cancer Res.

[B18] Vogel U, Laros I, Jacobsen NR, Thomsen BL, Bak H, Olsen A, Bukowy Z, Wallin H, Overvad K, Tjonneland A, Nexo BA, Raaschou-Nielsen O (2004). Two regions in chromosome 19q13.2-3 are associated with risk of lung cancer. Mutat Res.

[B19] Popanda O, Schattenberg T, Phong CT, Butkiewicz D, Risch A, Edler L, Kayser K, Dienemann H, Schulz V, Drings P, Bartsch H, Schmezer P (2004). Specific combinations of DNA repair gene variants and increased risk for non-small cell lung cancer. Carcinogenesis.

[B20] Zienolddiny S, Campa D, Lind H, Ryberg D, Skaug V, Stangeland L, Phillips DH, Canzian F, Haugen A (2006). Polymorphisms of DNA repair genes and risk of non-small cell lung cancer. Carcinogenesis.

[B21] Xing D, Tan W, Wei Q, Lin D (2002). Polymorphisms of the DNA repair gene XPD and risk of lung cancer in a Chinese population. Lung Cancer.

[B22] Liang G, Xing D, Miao X, Tan W, Yu C, Lu W, Lin D (2003). Sequence variations in the DNA repair gene XPD and risk of lung cancer in a Chinese population. Int J Cancer.

[B23] Hu Z, Xu L, Shao M, Yuan J, Wang Y, Wang F, Yuan W, Qian J, Ma H, Liu H, Chen W, Yang L, Jing G, Huo X, Chen F, Jin L, Wei Q, Wu T, Lu D, Huang W, Shen H (2006). Polymorphisms in the two helicases ERCC2/XPD and ERCC3/XPB of the transcription factor IIH complex and risk of lung cancer: a case-control analysis in a Chinese population. Cancer Epidemiol Biomarkers Prev.

[B24] Yin J, Vogel U, Ma Y, Guo L, Wang H, Qi R (2006). Polymorphism of the DNA repair gene ERCC2 Lys751Gln and risk of lung cancer in a northeastern Chinese population. Cancer Genet Cytogenet.

[B25] Hu Z, Wei Q, Wang X, Shen H (2004). DNA repair gene XPD polymorphism and lung cancer risk: a meta-analysis. Lung Cancer.

[B26] Vogel U, Overvad K, Wallin H, Tjonneland A, Nexo BA, Raaschou-Nielsen O (2005). Combinations of polymorphisms in XPD, XPC and XPA in relation to risk of lung cancer. Cancer Lett.

[B27] Au WW, Navasumrit P, Ruchirawat M (2004). Use of biomarkers to characterize functions of polymorphic DNA repair genotypes. Int J Hyg Environ Health.

[B28] Vodicka P, Kumar R, Stetina R, Sanyal S, Soucek P, Haufroid V, Dusinska M, Kuricova M, Zamecnikova M, Musak L, Buchancova J, Norppa H, Hirvonen A, Vodickova L, Naccarati A, Matousu Z, Hemminki K (2004). Genetic polymorphisms in DNA repair genes and possible links with DNA repair rates, chromosomal aberrations and single-strand breaks in DNA. Carcinogenesis.

[B29] Pavanello S, Pulliero A, Siwinska E, Mielzynska D, Clonfero E (2005). Reduced nucleotide excision repair and GSTM1-null genotypes influence anti-B[a]PDE-DNA adduct levels in mononuclear white blood cells of highly PAH-exposed coke oven workers. Carcinogenesis.

[B30] Neumann AS, Sturgis EM, Wei Q (2005). Nucleotide excision repair as a marker for susceptibility to tobacco-related cancers: a review of molecular epidemiological studies. Mol Carcinog.

[B31] Frosina G (2004). Commentary: DNA base excision repair defects in human pathologies. Free Radic Res.

[B32] Wilson DM, Sofinowski TM, McNeill DR (2003). Repair mechanisms for oxidative DNA damage. Front Biosci.

[B33] Vogel U, Nexo BA, Wallin H, Overvad K, Tjonneland A, Raaschou-Nielsen O (2004). No association between base excision repair gene polymorphisms and risk of lung cancer. Biochem Genet.

[B34] Hung RJ, Brennan P, Canzian F, Szeszenia-Dabrowska N, Zaridze D, Lissowska J, Rudnai P, Fabianova E, Mates D, Foretova L, Janout V, Bencko V, Chabrier A, Borel S, Hall J, Boffetta P (2005). Large-scale investigation of base excision repair genetic polymorphisms and lung cancer risk in a multicenter study. J Natl Cancer Inst.

[B35] Schneider J, Classen V, Bernges U, Philipp M (2005). XRCC1 polymorphism and lung cancer risk in relation to tobacco smoking. Int J Mol Med.

[B36] Zhang X, Miao X, Liang G, Hao B, Wang Y, Tan W, Li Y, Guo Y, He F, Wei Q, Lin D (2005). Polymorphisms in DNA base excision repair genes ADPRT and XRCC1 and risk of lung cancer. Cancer Res.

[B37] Kiyohara C, Takayama K, Nakanishi Y (2006). Association of genetic polymorphisms in the base excision repair pathway with lung cancer risk: a meta-analysis. Lung Cancer.

[B38] Ryk C, Kumar R, Thirumaran RK, Hou SM (2006). Polymorphisms in the DNA repair genes XRCC1, APEX1, XRCC3 and NBS1, and the risk for lung cancer in never- and ever-smokers. Lung Cancer.

[B39] Yin J, Vogel U, Ma Y, Qi R, Sun Z, Wang H (2007). The DNA repair gene XRCC1 and genetic susceptibility of lung cancer in a northeastern Chinese population. Lung Cancer.

[B40] Khanna KK, Jackson SP (2001). DNA double-strand breaks: signaling, repair and the cancer connection. Nat Genet.

[B41] David-Beabes GL, Lunn RM, London SJ (2001). No association between the XPD (Lys751G1n) polymorphism or the XRCC3 (Thr241Met) polymorphism and lung cancer risk. Cancer Epidemiol Biomarkers Prev.

[B42] Misra RR, Ratnasinghe D, Tangrea JA, Virtamo J, Andersen MR, Barrett M, Taylor PR, Albanes D (2003). Polymorphisms in the DNA repair genes XPD, XRCC1, XRCC3, and APE/ref-1, and the risk of lung cancer among male smokers in Finland. Cancer Lett.

[B43] Jacobsen NR, Raaschou-Nielsen O, Nexo B, Wallin H, Overvad K, Tjonneland A, Vogel U (2004). XRCC3 polymorphisms and risk of lung cancer. Cancer Lett.

[B44] Brownson RC, Alavanja MC, Hock ET, Loy TS (1992). Passive smoking and lung cancer in nonsmoking women. Am J Public Health.

[B45] Miller SA, Dykes DD, Polesky HF (1988). A simple salting out procedure for extracting DNA from human nucleated cells. Nucleic Acids Res.

[B46] Wolf FM (1986). Meta-analysis : quantitative methods for research synthesis.

[B47] Butkiewicz D, Rusin M, Enewold L, Shields PG, Chorazy M, Harris CC (2001). Genetic polymorphisms in DNA repair genes and risk of lung cancer. Carcinogenesis.

[B48] Spitz MR, Wu X, Wang Y, Wang LE, Shete S, Amos CI, Guo Z, Lei L, Mohrenweiser H, Wei Q (2001). Modulation of nucleotide excision repair capacity by XPD polymorphisms in lung cancer patients. Cancer Res.

[B49] Campbell CD, Ogburn EL, Lunetta KL, Lyon HN, Freedman ML, Groop LC, Altshuler D, Ardlie KG, Hirschhorn JN (2005). Demonstrating stratification in a European American population. Nat Genet.

[B50] Khan SG, Muniz-Medina V, Shahlavi T, Baker CC, Inui H, Ueda T, Emmert S, Schneider TD, Kraemer KH (2002). The human XPC DNA repair gene: arrangement, splice site information content and influence of a single nucleotide polymorphism in a splice acceptor site on alternative splicing and function. Nucleic Acids Res.

[B51] Sanyal S, Festa F, Sakano S, Zhang Z, Steineck G, Norming U, Wijkstrom H, Larsson P, Kumar R, Hemminki K (2004). Polymorphisms in DNA repair and metabolic genes in bladder cancer. Carcinogenesis.

[B52] Garcia-Closas M, Malats N, Real FX, Welch R, Kogevinas M, Chatterjee N, Pfeiffer R, Silverman D, Dosemeci M, Tardon A, Serra C, Carrato A, Garcia-Closas R, Castano-Vinyals G, Chanock S, Yeager M, Rothman N (2006). Genetic variation in the nucleotide excision repair pathway and bladder cancer risk. Cancer Epidemiol Biomarkers Prev.

[B53] Lee GY, Jang JS, Lee SY, Jeon HS, Kim KM, Choi JE, Park JM, Chae MH, Lee WK, Kam S, Kim IS, Lee JT, Jung TH, Park JY (2005). XPC polymorphisms and lung cancer risk. Int J Cancer.

[B54] Shen M, Berndt SI, Rothman N, Demarini DM, Mumford JL, He X, Bonner MR, Tian L, Yeager M, Welch R, Chanock S, Zheng T, Caporaso N, Lan Q (2005). Polymorphisms in the DNA nucleotide excision repair genes and lung cancer risk in Xuan Wei, China. Int J Cancer.

[B55] Qiao Y, Spitz MR, Shen H, Guo Z, Shete S, Hedayati M, Grossman L, Mohrenweiser H, Wei Q (2002). Modulation of repair of ultraviolet damage in the host-cell reactivation assay by polymorphic XPC and XPD/ERCC2 genotypes. Carcinogenesis.

[B56] Qiao Y, Spitz MR, Guo Z, Hadeyati M, Grossman L, Kraemer KH, Wei Q (2002). Rapid assessment of repair of ultraviolet DNA damage with a modified host-cell reactivation assay using a luciferase reporter gene and correlation with polymorphisms of DNA repair genes in normal human lymphocytes. Mutat Res.

[B57] Palli D, Russo A, Masala G, Saieva C, Guarrera S, Carturan S, Munnia A, Matullo G, Peluso M (2001). DNA adduct levels and DNA repair polymorphisms in traffic-exposed workers and a general population sample. Int J Cancer.

[B58] Hou SM, Falt S, Angelini S, Yang K, Nyberg F, Lambert B, Hemminki K (2002). The XPD variant alleles are associated with increased aromatic DNA adduct level and lung cancer risk. Carcinogenesis.

[B59] Tang D, Cho S, Rundle A, Chen S, Phillips D, Zhou J, Hsu Y, Schnabel F, Estabrook A, Perera FP (2002). Polymorphisms in the DNA repair enzyme XPD are associated with increased levels of PAH-DNA adducts in a case-control study of breast cancer. Breast Cancer Res Treat.

[B60] Chen S, Tang D, Xue K, Xu L, Ma G, Hsu Y, Cho SS (2002). DNA repair gene XRCC1 and XPD polymorphisms and risk of lung cancer in a Chinese population. Carcinogenesis.

[B61] (2004). Tobacco smoke and involuntary smoking. IARC Monogr Eval Carcinog Risks Hum.

[B62] Deutsch-Wenzel RP, Brune H, Grimmer G, Dettbarn G, Misfeld J (1983). Experimental studies in rat lungs on the carcinogenicity and dose-response relationships of eight frequently occurring environmental polycyclic aromatic hydrocarbons. J Natl Cancer Inst.

[B63] Hoffman (1993). Cigarette smoking and adenocarcinoma of the lung: the relevance of nicotine-derived nitrosamines. J Smoking Relat Disord.

[B64] Abdel-Rahman SZ, Soliman AS, Bondy ML, Omar S, El-Badawy SA, Khaled HM, Seifeldin IA, Levin B (2000). Inheritance of the 194Trp and the 399Gln variant alleles of the DNA repair gene XRCC1 are associated with increased risk of early-onset colorectal carcinoma in Egypt. Cancer Lett.

[B65] Duell EJ, Millikan RC, Pittman GS, Winkel S, Lunn RM, Tse CK, Eaton A, Mohrenweiser HW, Newman B, Bell DA (2001). Polymorphisms in the DNA repair gene XRCC1 and breast cancer. Cancer Epidemiol Biomarkers Prev.

[B66] Kuschel B, Auranen A, McBride S, Novik KL, Antoniou A, Lipscombe JM, Day NE, Easton DF, Ponder BA, Pharoah PD, Dunning A (2002). Variants in DNA double-strand break repair genes and breast cancer susceptibility. Hum Mol Genet.

[B67] Chacko P, Rajan B, Joseph T, Mathew BS, Pillai MR (2005). Polymorphisms in DNA repair gene XRCC1 and increased genetic susceptibility to breast cancer. Breast Cancer Res Treat.

[B68] Divine KK, Gilliland FD, Crowell RE, Stidley CA, Bocklage TJ, Cook DL, Belinsky SA (2001). The XRCC1 399 glutamine allele is a risk factor for adenocarcinoma of the lung. Mutat Res.

[B69] Park JY, Lee SY, Jeon HS, Bae NC, Chae SC, Joo S, Kim CH, Park JH, Kam S, Kim IS, Jung TH (2002). Polymorphism of the DNA repair gene XRCC1 and risk of primary lung cancer. Cancer Epidemiol Biomarkers Prev.

[B70] Winsey SL, Haldar NA, Marsh HP, Bunce M, Marshall SE, Harris AL, Wojnarowska F, Welsh KI (2000). A variant within the DNA repair gene XRCC3 is associated with the development of melanoma skin cancer. Cancer Res.

[B71] Stern MC, Umbach DM, van Gils CH, Lunn RM, Taylor JA (2001). DNA repair gene XRCC1 polymorphisms, smoking, and bladder cancer risk. Cancer Epidemiol Biomarkers Prev.

[B72] Olshan AF, Watson MA, Weissler MC, Bell DA (2002). XRCC1 polymorphisms and head and neck cancer. Cancer Lett.

[B73] Huang WY, Olshan AF, Schwartz SM, Berndt SI, Chen C, Llaca V, Chanock SJ, Fraumeni JF, Hayes RB (2005). Selected genetic polymorphisms in MGMT, XRCC1, XPD, and XRCC3 and risk of head and neck cancer: a pooled analysis. Cancer Epidemiol Biomarkers Prev.

[B74] Hung RJ, Hall J, Brennan P, Boffetta P (2005). Genetic polymorphisms in the base excision repair pathway and cancer risk: a HuGE review. Am J Epidemiol.

[B75] Matullo G, Palli D, Peluso M, Guarrera S, Carturan S, Celentano E, Krogh V, Munnia A, Tumino R, Polidoro S, Piazza A, Vineis P (2001). XRCC1, XRCC3, XPD gene polymorphisms, smoking and (32)P-DNA adducts in a sample of healthy subjects. Carcinogenesis.

[B76] Lindh AR, Rafii S, Schultz N, Cox A, Helleday T (2006). Mitotic defects in XRCC3 variants T241M and D213N and their relation to cancer susceptibility. Hum Mol Genet.

[B77] Araujo FD, Pierce AJ, Stark JM, Jasin M (2002). Variant XRCC3 implicated in cancer is functional in homology-directed repair of double-strand breaks. Oncogene.

[B78] Sancar A (1996). DNA excision repair. Annu Rev Biochem.

[B79] Wang XW, Vermeulen W, Coursen JD, Gibson M, Lupold SE, Forrester K, Xu G, Elmore L, Yeh H, Hoeijmakers JH, Harris CC (1996). The XPB and XPD DNA helicases are components of the p53-mediated apoptosis pathway. Genes Dev.

