# Supplementary material for: Polymorphisms in XPC, XPD, XRCC1, and XRCC3 DNA repair genes and lung cancer risk in a population of Northern Spain
Source: BMC Cancer. 2007 Aug 16;7:162. doi: 10.1186/1471-2407-7-162 (PMC2020474; doi:10.1186/1471-2407-7-162)
Supplement: Additional file 1 — Table 6 – Analysis of XPC PAT stratified by selected variables. This table shows the stratified analysis by selected variables of XPC PAT polymorphism [file 1471-2407-7-162-S1.doc]

**Table 6 - Analysis of *XPC* PAT stratified by selected variables**

|  | **Adjusted OR [95% IC]^*^** | | | | | | | | |  |
| --- | --- | --- | --- | --- | --- | --- | --- | --- | --- | --- |
| Variables | **-/-** | **Cases**  **n (%)** | **Controls**  **n (%)** | **+/-** | ***P*** | **Cases**  **n (%)** | **Controls**  **n (%)** | **+/+** | ***P*** | ***P* trend** |
| Smoking status  ETS exposed  Ever  Former  Current^a^ | 1.00  1.00  1.00  1.00 | 17 (48.6)  226 (47.1)  96 (43.2)  129 (51.2) | 67 (47.5)  192 (49.4)  111 (49.6)  72 (48.3) | 1.03 [0.41-2.55]  1.02 [0.75-1.37]  0.86 [0.57-1.30]  1.14 [0.71-1.81] | 0.953  0.908  0.488  0.591 | 6 (17.1)  95 (19.8)  42 (18.9)  49 (19.4) | 24 (17.0)  59 (15.2)  28 (12.5)  29 (19.5) | 1.05 [0.31-3.51]  **1.40 [0.94-2.08]**  1.59 [0.90-2.82]  1.06 [0.59-1.92] | 0.940  0.100  0.113  0.834 | 0.936  0.149  0.279  0.775 |
| Cumulative tobacco consumption^b^  Light  Moderate  Heavy | 1.00  1.00  1.00 | 13 (38.2)  57 (47.5)  154 (49.2) | 58 (47.9)  59 (49.2)  56 (46.7) | 0.59 [0.21-1.69]  1.12 [0.71-1.77]  1.19 [0.70-2.03] | 0.330  0.626  0.520 | 6 (17.6)  24 (20.0)  58 (18.5) | 15 (12.4)  19 (15.8)  23 (19.2) | 1.24 [0.31-5.05]  1.29 [0.73-2.28]  1.40 [0.66-3.00] | 0.761  0.379  0.382 | 0.905  0.378  0.352 |
| Cumulative tobacco consumption (only black) ^b^  Light  Moderate  Heavy | 1.00  1.00  1.00 | 9 (37.5)  37 (45.1)  124 (50.4) | 30 (46.9)  40 (54.0)  39 (48.1) | 0.33 [0.09-1.20]  0.84 [0.47-1.50]  1.33 [0.70-2.50] | 0.093  0.559  0.379 | 3 (12.5)  17 (20.7)  47 (19.1) | 8 (12.5)  10 (13.5)  16 (19.7) | 0.95 [0.14-6.38]  1.09 [0.54-2.20]  1.55 [0.62-3.87] | 0.961  0.806  0.350 | 0.377  0.897  0.379 |
| Family history of cancer  No  Lung cancer  Other cancers | 1.00  1.00  1.00 | 128 (47.1)  25 (43.9)  70 (47.9) | 155 (48.9)  23 (65.7)  75 (47.5) | 1.10 [0.73-1.64]  **0.30 [0.08-1.10]**  1.08 [0.58-2.00] | 0.644  **0.069**  0.806 | 51 (18.7)  9 (15.8)  34 (23.3) | 52 (16.4)  3 (8.6)  25 (15.8) | 1.21 [0.71-2.06]  1.49 [0.13-16.68]  1.42 [0.66-3.06] | 0.477  0.745  0.374 | 0.464  0.540  0.398 |
| Histologic type  Squamous cell carcinoma  Adenocarcinoma  Small cell carcinoma | 1.00  1.00  1.00 | 90 (43.3)  78 (51.3)  34 (41.0) | 259 (48.6)  259 (48.6)  259 (48.6) | 0.94 [0.62-1.41]  **1.52 [0.97-2.38]**  0.83 [0.48-1.44] | 0.757  **0.070**  0.515 | 46 (22.1)  32 (21.1)  15 (18.1) | 84 (15.8)  84 (15.8)  84 (15.8) | 1.44 [0.85-2.44]  **1.72 [0.97-3.04]**  1.09 [0.54-2.21] | 0.175  **0.064**  0.801 | 0.279  **0.043**  0.988 |

^*^ Odds ratios (ORs) adjusted by age, gender and cumulative tobacco consumption (in pack-years: ≤16.45, >16.45-53 and >53)

^a^ Former ≤ 1 year are included

^b^ Odds ratios adjusted by age and gender
