# Supplementary material for: Polymorphisms in XPC, XPD, XRCC1, and XRCC3 DNA repair genes and lung cancer risk in a population of Northern Spain
Source: BMC Cancer. 2007 Aug 16;7:162. doi: 10.1186/1471-2407-7-162 (PMC2020474; doi:10.1186/1471-2407-7-162)
Supplement: Additional file 2 — Table 7 – Analysis of XPD exon 10 stratified by selected variables. This table shows the stratified analysis by selected variables of XPD exon 10 (Asp312Asn) polymorphism [file 1471-2407-7-162-S2.doc]

**Table 7 - Analysis of *XPD* exon 10 stratified by selected variables**

|  | **Adjusted OR [95% IC]^*^** | | | | | | | | |  |
| --- | --- | --- | --- | --- | --- | --- | --- | --- | --- | --- |
| Variables | ***Asp/Asp*** | **Cases**  **n (%)** | **Controls**  **n (%)** | ***Asp/Asn*** | ***P*** | **Cases**  **n (%)** | **Controls**  **n (%)** | ***Asn/Asn*** | ***P*** | ***P* trend** |
| Smoking status  ETS exposed  Ever  Former  Current^a^ | 1.00  1.00  1.00  1.00 | 18 (51.4)  202 (42.1)  96 (43.2)  103 (40.9) | 64 (45.4)  164 (42.2)  93 (41.5)  63 (42.3) | 1.75 [0.72-4.24]  1.06 [0.80-1.40]  1.11 [0.75-1.65]  1.00 [0.65-1.54] | 0.213  0.693  0.597  0.987 | 4 (11.4)  51 (10.6)  23 (10.4)  27 (10.7) | 15 (10.6)  28 (7.2)  18 (8.0)  10 (6.7) | 2.58 [0.60-11.12]  **1.58 [0.96-2.60]**  1.36 [0.69-2.68]  1.72 [0.78-3.75] | 0.203  0.074  0.380  0.176 | 0.126  0.139  0.364  0.329 |
| Cumulative tobacco consumption^b^  Light  Moderate  Heavy | 1.00  1.00  1.00 | 13 (38.2)  47 (39.2)  137 (43.8) | 49 (40.5)  48 (40.0)  57 (47.5) | 1.11 [0.40-3.05]  0.98 [0.64-1.51]  0.87 [0.53-1.45] | 0.842  0.945  0.599 | 3 (8.8)  11 (9.2)  36 (11.5) | 11 (9.1)  9 (7.5)  7 (5.8) | 1.75 [0.37-8.31]  1.24 [0.59-2.62]  2.07 [0.74-5.75] | 0.484  0.573  0.165 | 0.544  0.718  0.476 |
| Cumulative tobacco consumption (only black)^b^  Light  Moderate  Heavy | 1.00  1.00  1.00 | 8 (33.3)  29 (35.4)  111 (45.1) | 29 (45.3)  30 (40.5)  36 (44.4) | 1.02 [0.30-3.50]  0.82 [0.48-1.40]  0.96 [0.53-1.76] | 0.976  0.476  0.906 | 2 (8.3)  7 (8.5)  26 (10.6) | 9 (14.1)  5 (6.8)  6 (7.4) | 1.03 [0.16-6.44]  0.96 [0.38-2.45]  0.96 [0.53-1.76] | 0.972  0.933  0.346 | 0.968  0.643  0.544 |
| Family history of cancer  No  Lung cancer  Other cancers | 1.00  1.00  1.00 | 126 (46.3)  17 (29.8)  56 (38.4) | 134 (42.3)  17 (48.6)  68 (43.0) | 1.22 [0.83-1.79]  0.35 [0.10-1.26]  0.81 [0.46-1.44] | 0.305  0.109  0.480 | 28 (10.3)  9 (15.8)  16 (11.0) | 26 (8.2)  2 (5.7)  13 (8.2) | 1.48 [0.77-2.85]  -  1.62 [0.60-4.36] | 0.241  -  0.336 | 0.241  0.621  0.751 |
| Histologic type  Squamous cell carcinoma  Adenocarcinoma  Small cell carcinoma | 1.00  1.00  1.00 | 80 (38.5)  67 (44.1)  41 (49.4) | 230 (43.1)  230 (43.1)  230 (43.1) | 0.78 [0.52-1.15]  1.10 [0.73-1.67]  1.34 [0.79-2.25] | 0.205  0.645  0.275 | 19 (9.1)  43 (8.1)  9 (10.8) | 43 (8.1)  43 (8.1)  43 (8.1) | 1.02 [0.52-2.03]  **1.88 [0.97-3.63]**  1.91 [0.80-4.52] | 0.942  **0.061**  0.142 | 0.503  0.116  0.111 |

^*^ Odds ratios (ORs) adjusted by age, gender and cumulative tobacco consumption (in pack-years: ≤16.45, >16.45-53 and >53)

^a^ Former ≤ 1 year are included

^b^ Odds ratios adjusted by age and gender
