# Supplementary material for: Polymorphisms in XPC, XPD, XRCC1, and XRCC3 DNA repair genes and lung cancer risk in a population of Northern Spain
Source: BMC Cancer. 2007 Aug 16;7:162. doi: 10.1186/1471-2407-7-162 (PMC2020474; doi:10.1186/1471-2407-7-162)
Supplement: Additional file 3 — Table 8 – Analysis of XRCC1 Arg399Gln stratified by selected variables. This table shows the stratified analysis by selected variables of XRCC1 Arg399Gln polymorphism [file 1471-2407-7-162-S3.doc]

**Table 8 - Analysis of *XRCC1* Arg399Gln stratified by selected variables**

|  | **Adjusted OR [95% IC]^*^** | | | | | | | | |  |
| --- | --- | --- | --- | --- | --- | --- | --- | --- | --- | --- |
| Variables | ***Arg/Arg*** | **Cases**  **n (%)** | **Controls**  **n (%)** | ***Arg/Gln*** | ***P*** | **Cases**  **n (%)** | **Controls**  **n (%)** | ***Gln/Gln*** | ***P*** | ***P* trend** |
| Smoking status  ETS exposed  Ever  Former  Current^a^ | 1.00  1.00  1.00  1.00 | 13 (37.1)  206 (42.9)  88 (39.6)  114 (45.2) | 68 (48.2)  165 (42.4)  100 (44.6)  58 (38.9) | 0.68 [0.28-1.64]  1.01 [0.75-1.35]  0.79 [0.52-1.18]  1.32 [0.84-2.06] | 0.387  0.940  0.246  0.230 | 4 (11.4)  71 (14.8)  30 (13.5)  41 (16.3) | 21 (14.9)  62 (15.9)  33 (14.7)  27 (18.1) | 0.53 [0.14-1.92]  0.94 [0.63-1.40]  0.78 [0.44-1.39]  1.04 [0.58-1.87] | 0.334  0.747  0.404  0.894 | 0.265  0.812  0.266  0.632 |
| Cumulative tobacco consumption^b^  Light  Moderate  Heavy | 1.00  1.00  1.00 | 11 (32.3)  55 (45.8)  131 (41.8) | 52 (43.0)  49 (40.8)  55 (45.8) | 0.66 [0.22-1.98]  1.13 [0.73-1.76]  0.70 [0.41-1.18] | 0.458  0.582  0.183 | 9 (26.5)  14 (11.7)  48 (15.3) | 20 (16.5)  22 (18.3)  17 (14.2) | 1.62 [0.47-5.56]  0.67 [0.36-1.24]  1.11 [0.52-2.36] | 0.444  0.203  0.790 | 0.629  0.397  0.812 |
| Cumulative tobacco consumption (only black)^b^  Light  Moderate  Heavy | 1.00  1.00  1.00 | 7 (29.2)  34 (41.5)  104 (42.3) | 35 (54.7)  33 (44.6)  36 (44.4) | 0.33 [0.08-1.35]  0.86 [0.50-1.47]  0.86 [0.47-1.60] | 0.124  0.585  0.643 | 7 (29.2)  8 (9.8)  35 (14.2) | 8 (12.5)  9 (12.2)  10 (12.3) | 2.51 [0.52-12.05]  0.73 [0.32-1.65]  1.37 [0.54-3.50] | 0.250  0.449  0.509 | 0.555  0.412  0.728 |
| Family history of cancer  No  Lung cancer  Other cancers | 1.00  1.00  1.00 | 110 (40.4)  29 (50.9)  62 (42.5) | 138 (43.5)  19 (54.3)  62 (39.4) | 0.80 [0.54-1.19]  1.51 [0.44-5.11]  0.82 [0.45-1.48] | 0.279  0.511  0.515 | 31 (11.4)  8 (14.0)  26 (17.8) | 55 (17.3)  2 (5.7)  21 (13.3) | **0.57 [0.33-0.98]**  5.72 [0.49-66.13]  1.48 [0.66-3.30] | **0.042**  0.162  0.343 | **0.041**  0.168  0.567 |
| Histologic type  Squamous cell carcinoma  Adenocarcinoma  Small cell carcinoma | 1.00  1.00  1.00 | 94 (45.2)  61 (40.1)  37 (44.6) | 234 (43.9)  234 (43.9)  234 (43.9) | 0.95 [0.64-1.41]  0.74 [0.49-1.14]  1.06 [0.62-1.82] | 0.795  0.177  0.821 | 27 (13.0)  24 (15.8)  13 (15.7) | 82 (15.4)  82 (15.4)  82 (15.4) | 0.81 [0.46-1.41]  0.99 [0.56-1.74]  1.01 [0.49-2.09] | 0.795  0.964  0.982 | 0.480  0.619  0.9927 |

^*^ Odds ratios (ORs) adjusted by age, gender and cumulative tobacco consumption (in pack-years: ≤16.45, >16.45-53 and >53)

^a^ Former ≤ 1 year are included

^b^ Odds ratios adjusted by age and gender
