# Supplementary material for: Polymorphisms in XPC, XPD, XRCC1, and XRCC3 DNA repair genes and lung cancer risk in a population of Northern Spain
Source: BMC Cancer. 2007 Aug 16;7:162. doi: 10.1186/1471-2407-7-162 (PMC2020474; doi:10.1186/1471-2407-7-162)
Supplement: Additional file 4 — Table 9 – Analysis of XRCC3 Thr241Met stratified by selected variables. This table shows the stratified analysis by selected variables of XRCC3 Thr241Met polymorphism [file 1471-2407-7-162-S4.doc]

**Table 9 - Analysis of *XRCC3* Thr241Met stratified by selected variables**

|  | **Adjusted OR [95% IC]^*^** | | | | | | | | |  |
| --- | --- | --- | --- | --- | --- | --- | --- | --- | --- | --- |
| Variables | ***Thr/Thr*** | **Cases**  **n (%)** | **Controls**  **n (%)** | ***Thr/Met*** | ***P*** | **Cases**  **n (%)** | **Controls**  **n (%)** | ***Met/Met*** | ***P*** | ***P* trend** |
| Smoking status  ETS exposed  Ever  Former  Current^a^ | 1.00  1.00  1.00  1.00 | 15 (57.7)  169 (44.9)  72 (41.2)  96 (48.2) | 55 (49.1)  140 (43.9)  83 (43.5)  51 (44.0) | 1.43 [0.53-3.89]  1.01 [0.73-1.39]  0.88 [0.55-1.39]  1.25 [0.76-2.04] | 0.482  0.968  0.577  0.375 | 3 (11.5)  47 (12.5)  24 (13.9)  23 (11.6) | 11 (9.8)  49 (15.4)  36 (18.8)  13 (11.2) | 1.87 [0.37-9.45]  0.77 [0.48-1.22]  0.63 [0.34-1.17]  1.14 [0.53-2.46] | 0.446  0.266  0.141  0.742 | 0.379  0.385  0.158  0.510 |
| Cumulative tobacco consumption^b^  Light  Moderate  Heavy | 1.00  1.00  1.00 | 6 (23.1)  47 (52.2)  111 (44.8) | 46 (45.5)  45 (44.1)  40 (43.0) | 0.60 [0.17-2.09]  1.03 [0.63-1.67]  1.06 [0.57-1.97] | 0.420  0.903  0.845 | 7 (26.9)  8 (8.9)  31 (12.5) | 15 (14.8)  19 (18.6)  12 (12.9) | 1.68 [0.45-6.30]  1.03 [0.63-1.67]  0.92 [0.38-2.24] | 0.443  0.154  0.864 | 0.599  0.282  0.955 |
| Cumulative tobacco consumption (only black)^b^  Light  Moderate  Heavy | 1.00  1.00  1.00 | 6 (31.6)  33 (54.1)  89 (45.6) | 23 (40.0)  30 (45.4)  28 (43.7) | 1.32 [0.29-6.01]  1.04 [0.57-1.90]  1.16 [0.56-2.39] | 0.716  0.887  0.694 | 5 (26.3)  6 (9.8)  24 (12.3) | 9 (15.5)  10 (15.1)  8 (12.5) | 2.54 [0.45-14.40]  0.94 [0.39-2.27]  0.84 [0.29-2.45] | 0.292  0.895  0.753 | 0.305  0.963  0.945 |
| Family history of cancer  No  Lung cancer  Other cancers | 1.00  1.00  1.00 | 102 (47.2)  19 (42.2)  52 (45.2) | 116 (45.8)  9 (37.5)  62 (44.9) | 1.04 [0.67-1.61]  0.49 [0.12-1.98]  1.51 [0.69-2.87] | 0.870  0.318  0.207 | 7 (26.9)  8 (8.9)  31 (12.5) | 15 (14.8)  19 (18.6)  12 (12.9) | 0.72 [0.38-1.36]  0.39 [0.05-3.31]  2.19 [0.84-5.68] | 0.309  0.391  0.108 | 0.452  0.282  0.077 |
| Histologic type  Squamous cell carcinoma  Adenocarcinoma  Small cell carcinoma | 1.00  1.00  1.00 | 69 (43.7)  57 (48.3)  28 (41.8) | 196 (45.2)  196 (45.2)  196 (45.2) | 0.87 [0.55-1.36]  1.19 [0.74-1.92]  0.91 [0.49-1.67] | 0.536  0.475  0.758 | 7 (26.9)  8 (8.9)  31 (12.5) | 15 (14.8)  19 (18.6)  12 (12.9) | **0.47 [0.23-1.00]**  1.14 [0.58-2.24]  1.26 [0.57-2.77] | **0.049**  0.711  0.570 | **0.072**  0.579  0.711 |

^*^ Odds ratios (ORs) adjusted by age, gender and cumulative tobacco consumption (in pack-years: ≤16.45, >16.45-53 and >53)

^a^ Former ≤ 1 year are included

^b^ Odds ratios adjusted by age and gender
